# Sex-based differences and aging in tactile function loss in persons with type 2 diabetes

**DOI:** 10.1371/journal.pone.0242199

**Published:** 2020-11-12

**Authors:** Stacey L. Gorniak, Nereyda Ochoa, Lauren I. Gulley Cox, Aisha Khan, Sahifah Ansari, Beatriz Thames, Haley Ray, Yoshimi F. Lu, Hidetaka Hibino, Nikita Watson, Patrick M. Dougherty

**Affiliations:** 1 Department of Health and Human Performance, University of Houston, Houston, TX, United States of America; 2 Department of Pain Medicine Research, University of Texas M.D. Anderson Cancer Center, Houston, TX, United States of America; Suez Canal University Faculty of Medicine, EGYPT

## Abstract

**Background:**

Recent evidence of significant sex-based differences in the presentation of Type 2 Diabetes Mellitus (DM) and its complications has been found in humans, which may contribute to sex-based differences in reduced functionality and quality of life. Some functionality, such as tactile function of the hands, has significant direct impact on quality of life. The purpose of the current study was to explore the impact of DM and sex on tactile function, with consideration of variability in health state measures.

**Research design and methods:**

A case-control single time point observational study from 2012–2020 in an ethnically diverse population-based community setting. The sample consists of 132 adult individuals: 70 independent community dwelling persons with DM (PwDM) and 62 age- and sex-matched controls (42 males and 90 females in total). The Semmes-Weinstein monofilament test was used to evaluate tactile sensation of the hands.

**Results:**

Tactile sensation thresholds were adversely impacted by sex, age, degree of handedness, high A_1c_, diagnosis of DM, and neuropathy. Overall, strongly right-handed older adult males with poorly controlled DM and neuropathy possessed the poorest tactile discrimination thresholds. When self-identified minority status was included in a secondary analysis, DM diagnosis was no longer significant; negative impacts of age, neuropathy, degree of handedness, and high A_1c_ remained significant.

**Conclusions:**

The data indicate significant impacts of male sex, age, degree of handedness, self-identified minority status, and metabolic health on the development of poor tactile sensation. This combination of modifiable and non-modifiable factors are important considerations in the monitoring and treatment of DM complications.

## Introduction

Over 30 million individuals in the United States (9.4%) are currently living with Type 2 Diabetes Mellitus (DM) [1]. Persons with DM (PwDM) experience declines in hand and finger sensorimotor function as compared to healthy individuals [2–5]; however, self-awareness of these changes is low [2, 5]. Reduced functional hand use has been associated with a loss of independent living and reduced quality of life [6]. Tactile sensitivity loss due to peripheral neuropathy (PN) has been implicated as the primary cause of both sensory and motor deficits in the hands and feet of PwDM [7–10]; however, our recent work has demonstrated that motor changes in PwDM occur independent of such tactile impairment, and appear to be unrelated to disease duration and severity [2–5, 11]. Our data indicate other physiological factors such as vascular dysfunction and functional cortical changes as the underlying mechanism for sensorimotor changes in PwDM [2–5, 11–14]. Due to the confluence of multiple systemic changes in the bodies of PwDM, the contribution of multiple systems−including sex hormone changes and impaired global hemodynamic function−to sensorimotor dysfunction prior to PN diagnosis in PwDM is fully plausible.

Recently, our group found no differences in tactile function of postmenopausal female PwDM as compared to age- and sex-matched controls during the assessment of upper extremity muscle hemodynamics [13]. This finding was intriguing as abnormal hemodynamic function of upper extremity muscle was found in PwDM and supports vascular dysfunction as a potential source of global sensorimotor changes in PwDM [10, 15], particularly in females as females have a higher overall risk of cardiovascular complications with age; however, the lack of between group tactile function differences challenged this point of view. Recent evidence suggests differential presentation of DM and its complications between the sexes−particularly with increased age [1, 16, 17]−potentially due to the anti-inflammatory, cardio-protective, and neuro-protective properties of testosterone [18]. Given that the dataset included only females, it is possible that sex hormones play a significant role in the maintenance or degradation of tactile function in conjunction with DM and aging.

In support of potential sex-based differences in sensory function and aging, some sensory differences have been found to exist between males and females (e.g., proprioception) [19, 20]. Additionally, it has been reported that females exhibit generation of more spinal excitatory potentials as compared to males [20]. It is not clear if these sex-based differences in sensory function persist, are exacerbated by, are or mitigated by the development and progression of DM.

In particular, older adult females are more negatively impacted by risks and complications associated with DM; which are linked to metabolic and sex-hormone changes during the transition from perimenopause to menopause [21]. As testosterone levels begin to decrease prior to perimenopause, the risk for development of cardiovascular disease (CVD), DM, increased adiposity, and systemic inflammation is enhanced. It is speculated that reduced testosterone and its anti-inflammatory effects in combination with the absence of neuroprotective estrogen in postmenopausal females negatively impact hemodynamic response, leading to worsened overall health outcomes for older adult females [22]. However, it is also known that PN occurs more frequently and earlier in males than in females [23–26]. Although the rate of progression to PN is comparable between sexes, significant sex-based differences in response to DM prevention interventions exists and resultant persistent sex-differences in DM comorbidities abound [27].

In order to explore the potential interaction of DM and sex specifically on tactile function, we have compiled tactile function assessment and health state information data for all studies performed in our laboratory from 2012 through 2020. The purpose of this compilation was to examine the data for impacts of sex, aging, PN, and measures of metabolic health (e.g., glycated hemoglobin, A_1c_) on tactile function. *A priori*, based on reports of increased risk of cardiovascular complications in female PwDM in the evidence base, we hypothesized that females would have worsened tactile function measures as compared to males, with negative impacts of increased age, PN diagnosis, and worsened metabolic health all contributing beyond sex-related impacts. Such findings would reinforce the evidence of sex-based differences in DM presentation in humans, which may directly contribute to sex-based differences in reduced functionality and quality of life with DM [28].

## Methods

### Participants

One hundred and thirty-two (132) adult individuals took part in this case-control single time point observational study from 2012–2020 in Houston, TX, USA. This sample consists of 70 independent community dwelling PwDM and 62 age- and sex-matched independent community dwelling controls (42 males and 90 females in total). A total of 27 cases of PN within the sample were found (20.7%). A total of 61 participants (46.2%) self-identified as either black/African American or Hispanic/Latino (henceforth referred to as minority persons). Average characteristics for study participants can be found in Table 1. Handedness was assessed by the Edinburgh Inventory [29], ranging from a laterality quotient (LQ) of –100 (strong left-handedness) to +100 (strong right-handedness). All study participants were right-handed based on an LQ of > +40 [29]. Study participants were excluded if they reported a history of neurological and/or musculoskeletal disorders such as: Parkinson disease, Huntington’s disease, polio, multiple sclerosis, stroke, traumatic brain injury, carpal tunnel syndrome, rheumatoid arthritis, Monoclonal Gammopathy of Undetermined Significance (MGUS), Paraproteinemic Demyelinating Neuropathy (PDN), Myasthenia Gravis, amputation, or other known hereditary or compression neuropathies. In accordance with the Declaration of Helsinki, participants provided written informed consent according to the regulations established by the Institutional Review Board at the University of Houston. The University of Houston Institutional Review Board approved all study procedures.

**Table 1:** Demographic description for study participants.

	PwDM		Controls	
	*M*	*F*	*M*	*F*
N	22	48	20	42
Age (years)	64.6 ± 9.0	63.0 ± 6.3	62.9 ± 6.8	63.5 ± 7.1
BMI (kg/m^2^)	31.0 ± 6.7	30.7 ± 7.0	27.1 ± 3.3	27.9 ± 7.1
A_1c_ (%)	7.8 ± 1.6	6.5 ± 1.5	5.3 ± 0.2	5.4 ± 0.4
A_1c_ (mmol/mol)	62 ± 17	48 ± 16	34 ± 3	36 ± 4
LQ	95 ± 10	97 ± 7	94 ± 8	97 ± 8
SYS (mmHg)	146 ± 17	140 ± 23	137 ± 12	140 ± 21
DIA (mmHg)	83 ± 13	82 ± 14	81 ± 10	83 ± 14
# cases PN	7 (5.4%)	17 (13.1%)	0 (0%)	3 (2.3%)
# self-identify as minority	9 (6.9%)	34 (25.8%)	1 (0.1%)	17 (13.1%)

Values are mean ± SD or count (% of entire sample). Entire sample is 132 participants. A_1c_ = glycated hemoglobin, BMI = body mass index, DIA = diastole, F = female, LQ = laterality quotient, M = male, PN = peripheral neuropathy, PwDM = person with Type 2 Diabetes, SYS = systole.

### Health status information

Glycated hemoglobin (A_1c_) values were assessed for all study participants on site. A_1c_ values were assessed using a commercially available point of care evaluation kit (A_1c_ Now+, PTS Diagnostics, Indianapolis, IN, USA). Blood pressure was measured for 113 of the study participants using a commercially available device (Omron Intellisense 10 series Blood Pressure Monitor, Model BP785, Bannockburn, IL, USA); blood pressure was not measured in our first study of the series in 2012. The presence of peripheral neuropathy (PN status) was determined by abnormalities on either clinical examination or EMG/NCV testing (per physician).

### Sensory evaluations

The Semmes-Weinstein monofilament test was used to evaluate tactile sensation of the hands. Monofilament testing sites included the following sites (and respective nerves): tip of the thumb/digit 1 (median nerve), the tip of digit 5 (ulnar nerve), and dorsal aspect of the thumb (radial nerve). During the test, participants kept their eyes closed and verbally indicated if and where they perceived monofilament touch. The monofilament size was increased in an ascending manner from the smallest monofilament unit until the subject was able to detect its touch a minimum of 2 times at the same location.

### Statistical analyses

**Fig 1:**
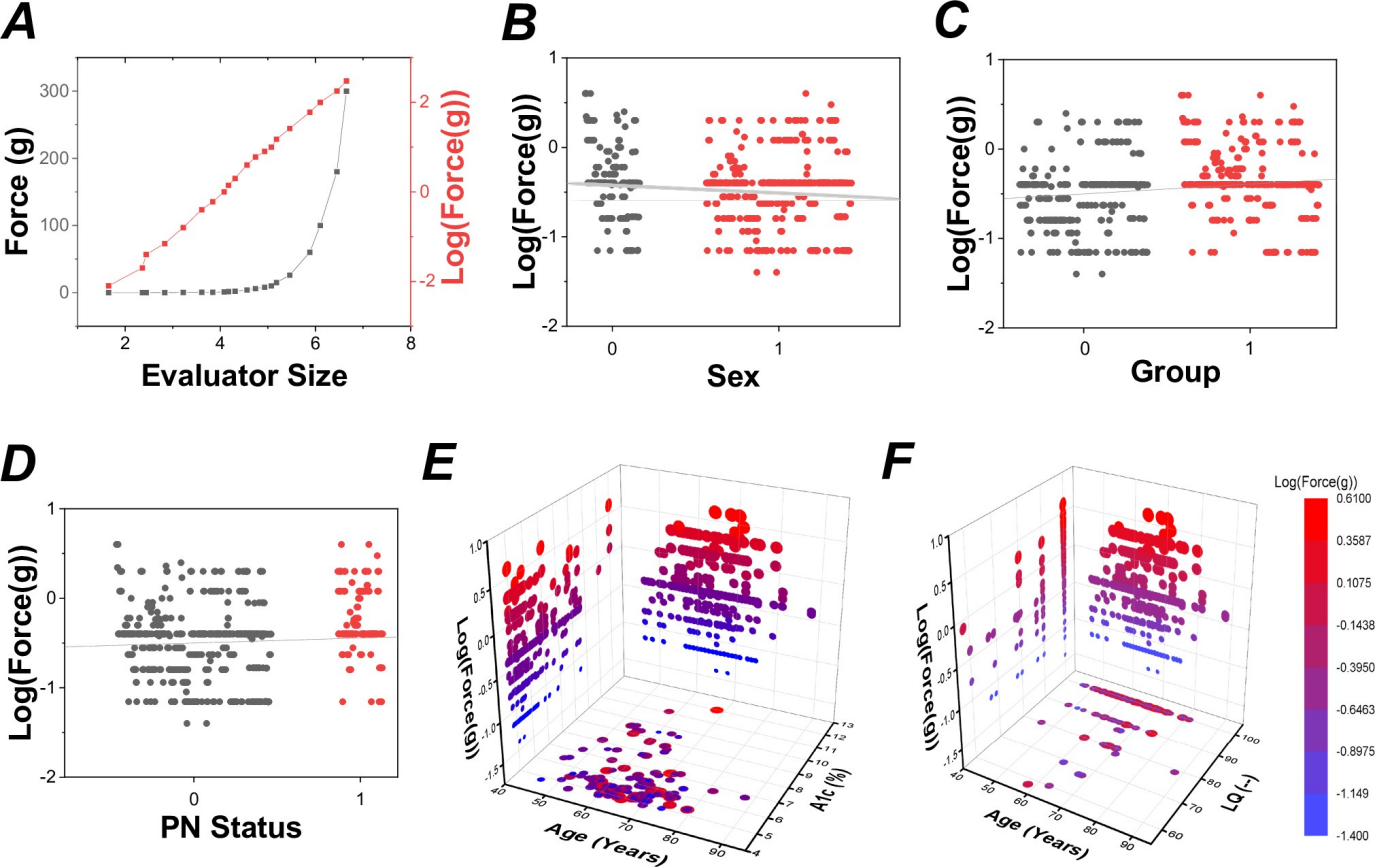
Monofilament data. **A:** Relationship between monofilament size and associated force sensitivity thresholds (in g). Monofilament data were log transformed (Log_10_(Force(g)) due to non-linearity inherent to measurement scale. **B:** Relationship between tactile threshold and *Sex*; 0 = male, 1 = female. The shaded gray region denotes the 95% confidence interval about the mean value. **C:** Relationship between tactile threshold and *Group*; 0 = control, 1 = PwDM. The shaded gray region denotes the 95% confidence interval about the mean value. **D:** Relationship between tactile threshold and *PN status*; 0 = no history, 1 = history of PN. The shaded gray region denotes the 95% confidence interval about the mean value. **E:** Relationship among tactile threshold, chronological age, and glycated hemoglobin (A_1c_). The projection of the 3D data set is shown for each plane of interest. **F:** Relationship among tactile threshold, chronological age, and laterality quotient (LQ). The projection of the 3D data set is shown for each plane of interest.

Data were compared between groups using mixed model regressions and subsequent analyses of covariance (ANCOVAs) via SPSS 25 (IBM Corporation, Armonk, NY, USA) and Minitab 17 (Minitab LLC, State College, PA, USA). Monofilament data were log transformed (Log_10_(Force(g)) due to non-linearity, illustrated in Fig 1A. Between-subject categorical factors for Log_10_(Force(g)) measures were *Group* (two levels: PwDM and age- and sex-matched controls) and *Sex* (two levels: male and female). Evaluation of monofilament testing site (e.g., median, radial, and ulnar nerves) as well as hand (right versus left) did not produce significant results in our analyses; thus, data are averaged across testing site and hand in the reported results. Evaluation of health state covariates was done to control for health state variability, both within and across the two sample groups. Health state covariates initially considered were: age, A_1c_, systole, diastole, disease duration (Duration), body mass index (BMI), PN status (via indicator variable), laterality quotient (LQ), and self-identification of belonging to a minority group (black/African American or Hispanic/Latino, via indicator variable). Covariates were selected via Automatic Linear Modeling (ALM) using forward stepwise selection functions in SPSS. In the event of significant covariates determined via ALM in SPSS and mixed regression/ANCOVA in Minitab, follow-up correlation analyses were performed between the health state covariate and tactile sensory function in Minitab.

## Results

### Tactile evaluation

Significant differences in tactile detection thresholds were found between categorical values in *Group* (F_1,616_ = 7.42, p < 0.01), *Sex* (F_1,616_ = 12.20, p < 0.001), and positive *PN status* (F_1,616_ = 20.31, p < 0.001); such that PwDM, males, and those with a history of PN had worsened detection thresholds, as shown in Fig 1B–1D. A *Group x Sex* interaction was not found. Tactile detection thresholds were further exacerbated by: increased *Age* (F_1,616_ = 7.23, p < 0.01), larger *LQ* values (F_1,616_ = 10.69, p < 0.001), and higher *A*_*1c*_ (F_1,616_ = 8.85, p < 0.005). A significant influence of *Duration* (F_1,616_ = 4.75, p < 0.05) was also found. A *Group x Sex* interaction was not found. Fig 1E and 1F illustrate these relationships. Coefficients of the full regression equation for Log_10_(Force(g)) can be found in Table 2.

**Table 2:** Original and secondary regression equation details for Log_10_(Force(g)).

*Factor*	*Coefficient*	*t-value*	*p-value*	*Coding Notes*
***Original Regression Model***				
Age (years)	0.00585	2.69	< 0.01	N/A
A_1c_ (%)	0.0507	2.97	< 0.005	N/A
Duration (months)	-0.000718	-2.18	< 0.05	N/A
Group	0.1031	2.72	< 0.01	0 = Control
				1 = PwDM
LQ	0.0064	3.27	< 0.001	> +40 (right-handed)
PN status	0.1765	4.51	< 0.001	0 = No history of PN
				1 = PN diagnosis
Sex	-0.1203	-3.49	< 0.001	0 = Male
				1 = Female
Regression constant	-1.726	-6.68	< 0.001	N/A
***Secondary Regression Model***				
Age (years)	0.00657	3.03	< 0.005	N/A
A_1c_ (%)	0.0455	2.68	< 0.01	N/A
Duration (months)	-0.000644	-1.97	0.05	N/A
LQ	0.00613	3.15	< 0.005	> +40 (right-handed)
Minority status	0.1181	3.41	< 0.001	0 = Does not identify as minority person
				1 = Does identify as minority person
PN status	0.1989	5.05	< 0.001	0 = No history of PN
				1 = PN diagnosis
Sex	-0.1635	-4.49	< 0.001	0 = Male
				1 = Female
Regression constant	-1.719	-6.70	< 0.001	N/A

A_1c_ = glycated hemoglobin, LQ = laterality quotient, N/A = not applicable, PN = peripheral neuropathy, PwDM = person with Type 2 Diabetes.

As incidence of DM is higher in minority persons, we explored the impact of self-declared minority status on Log_10_(Force(g)), using the same statistical approach. When *Minority status* (F_1,615_ = 11.63, p < 0.001) was included in the model, the *Group* effect was no longer significant and thus omitted from the secondary regression model. Significant deleterious impacts of male *Sex* (F_1,615_ = 20.15, p < 0.001), positive *PN status* (F_1,615_ = 25.51, p < 0.001), increased *Age* (F_1,615_ = 9.19, p < 0.001), larger *LQ* values (F_1,615_ = 9.95, p < 0.005), and higher *A*_*1c*_ (F_1,615_ = 7.19, p < 0.01) persisted in the secondary model. The influence of *Duration* (F_1,615_ = 3.87, p = 0.05) was also found to be at significance. A *Group x Sex* interaction was not found. Coefficients of the secondary regression equation for Log_10_(Force(g)) can be found in Table 2.

Simple bivariate correlation values of Log_10_(Force(g)) and variables of interest can be found in Table 3.

**Table 3:** Bivariate regression values for Log_10_(Force(g)) and variables of interest across the full data set.

*Factor*	*r*	*p-value*
Age (years)	0.075	0.056
A_1c_ (%)	0.221	< 0.001
Duration (months)	0.094	< 0.05
Minority status	0.117	< 0.005
Group	0.225	< 0.001
LQ	0.125	< 0.005
PN status	0.233	< 0.001
Sex	-0.144	< 0.001

A_1c_ = glycated hemoglobin, LQ = laterality quotient, PN = peripheral neuropathy.

Assessment of data from only PwDM indicated significant differences in tactile detection thresholds in persons with positive *PN status* (F_1,261_ = 15.12, p < 0.001), but not *Sex*. Tactile detection thresholds were further exacerbated by high *Diastole* values (F_1,261_ = 19.20, p < 0.001). Coefficients of the PwDM specific regression equation for Log_10_(Force(g)) can be found in Table 4. Posthoc inclusion of *Diastole* in the primary and secondary models for the entire data set as a single factor and as an interactive factor did not produce significant results.

**Table 4:** PwDM specific regression equation details for Log_10_(Force(g)).

*Factor*	*Coefficient*	*t-value*	*p-value*	*Coding Notes*
***PwDM Regression Model***				
Diastole (mmHg)	0.00679	4.38	< 0.001	N/A
PN status	0.1817	3.89	< 0.001	0 = No history of PN
				1 = PN diagnosis
Regression constant	-1.059	-8.27	< 0.001	N/A

PN = peripheral neuropathy.

## Discussion

The purpose of the current study was to explore the potential influence of DM and sex on tactile function, with consideration of variability in health state measures (e.g., PN diagnosis and A_1c_) in the statistical models. This study is a step towards better understanding sex-based differences in DM complications with aging. *A priori*, based on increased risk of cardiovascular complications in female PwDM in the evidence base, we hypothesized that females will have worsened tactile function measures as compared to males, with negative impacts of increased age, PN diagnosis, and worsened metabolic health all contributing beyond sex-related impacts. Our data indicate an opposite finding to our hypothesis with respect to sex-based differences in tactile function, yet provide evidence of significant impacts due to age, degree of handedness (via laterality quotient), self-identified minority status, and metabolic health. In the following paragraphs, we discuss sex-based differences in tactile function with respect to the evidence base, potential non-modifiable bases for these differences, and implications for both clinicians and patients.

### Influence of sex on sensory function and cortical structure/function

Contrary to our hypothesis, the data indicate general worsened tactile function in adult males as compared to adult females, independent of chronological age. Tactile sensation thresholds were additionally adversely impacted by chronological age and diagnosis of DM and PN, such that older adult males with DM and PN possessed the poorest tactile discrimination thresholds. This is consistent with reports of higher sensory sensitivity in healthy adult females without DM [30, 31] as well as reports of more frequent and earlier presentation of PN in males with DM [23–26]. While the presence of DM and PN were the most significant factors for reduce tactile function in PwDM, sex-based differences existed when the entire sample was considered, suggesting baseline sex-differences in tactile function. The root of these sex-based differences may stem from several sources. While testosterone may generally be considered anti-inflammatory, cardio-protective, and neuro-protective [18], these effects may not directly impact on peripheral nerve function. Instead, estrogen, estrogen signaling, and other androgens may play a larger role in the maintenance of peripheral nerves [32, 33]. Thus, it is possible that hormone therapy could be a potential pathway to preserving peripheral nerve function in males of advanced age prior to development of DM.

Additionally, evidence exists for sexual dimorphism and asymmetry of the cortex [33], indicating a confluence of central and peripheral impacts on tactile function. This sexual dimorphism presents as less cortical asymmetry (i.e., less lateralization) in both structure and function in females [30, 33, 34]. This presentation has been found to vary with handedness [30, 33, 34], consistent with our results. Within this specific area of research, it is not clear as to which of the sex hormones are specifically responsible for the dimorphism; as the complex interaction and impact of testosterone, androgens, and other confounding physiological factors have shown inconsistent trends [33].

Further support for sex-based differences in neurological function via the cortex arises from more mechanistic investigations of cortical dimorphism. Specifically, males are found to have a larger number of cortical neurons; however, these cortical neurons are generally smaller in size as compared to females [35]. It is possible that with aging, these smaller neurons may be at higher risk for degradation in males, given the recent report of persistent metabolic youth in the aging female brain as compared to males [36]. This chronological metabolic difference may also explain the earlier presentation and presence of neurological symptoms (e.g., PN) in males with DM [23–25, 37].

### (Non-)modifiable factors to tactile function and implications for clinicians and patients

The findings of the present study provide insight into a combination of modifiable and non-modifiable factors for clinicians to consider when treating complications of DM. The data reinforce the known importance of several factors associated with DM prevention such as controlling A_1c_ (glycated hemoglobin) and development of DM. Consistent with current clinical practice, prevention of DM, control of blood pressure, and control of long-term blood sugar across the lifespan are thus emphasized as the most modifiable factors leading to DM complications, including development of diabetic PN. These factors are so significant that they overtook other factors (including sex) contributing to tactile sensation loss in persons diagnosed with DM in the current study. It is essential to control these modifiable factors to prevent irreversible sensory loss in PwDM. Continued monitoring for DM while pursuing preventive measures for DM development by both the patient and clinician are strongly encouraged [38]. The preventive and treatment implications for patients are significant, as patients need to be aware of their control over the development of DM, its myriad complications, and the severity of potential long-term irreversible outcomes of DM. Reinforcement of this concept is critical to preserving long term peripheral nerve function.

The data also indicate a number of non-modifiable factors—such as chronological age, sex, and minority status—as significant in the development of tactile function loss as a DM complication. Despite the non-modifiable nature of these factors, they should be considered as risk factors in monitoring for DM and its complications [38]. Given the increased incidence of DM in minority populations, the development of significant tactile function loss in minority persons in the current sample reinforces the current clinical practice of additional medical monitoring for DM and its complications in persons who self-identify as a minority person.

Last, factors such as degree of handedness/laterality and sex-hormones also played a role in the current findings. There is significant interplay between these two factors, such that handedness/laterality is connected to sexual dimorphism [39, 40]. There is some evidence that handedness may be modifiable in the prenatal and early postnatal timeframes [40]; however, this is not a practical modifiable factor for clinicians treating older adults with DM. While there is some evidence of handedness being linked to cultural hand use and learning, the physiological roots of handedness/laterality typically minimize these social impacts [39]. Note that the study sample did not contain participants who were stroke survivors or persons with amputation, as the cortical plasticity associated with both conditions confounds sensorimotor function and functional hand use in both populations [41, 42].

## Conclusions

The purpose of the current study was to explore the potential interaction of DM and sex on tactile function, with consideration of variability in health state measures. The data indicate significant impacts of sex, age, degree of handedness/laterality, self-identified minority status, and metabolic health on the development of poor tactile sensation. We have discussed the sex-based differences in tactile function with respect to the evidence base, potential non-modifiable bases for these differences, and implications for both clinicians and patients.
